# The complete mitochondrial genome of *Oreolalax lichuanensis* (Amphibia, Anura, Megophryidae)

**DOI:** 10.1080/23802359.2016.1143340

**Published:** 2016-10-03

**Authors:** Gang Wang, Shouhong Wang, Xixi Liang, Feng Xie, Jianping Jiang, Bin Wang

**Affiliations:** a Chengdu Institute of Biology, Chinese Academy of Sciences, Chengdu, China;; b University of Chinese Academy of Sciences, Beijing, China

**Keywords:** Megophryidae, mitogenome, *Oreolalax lichuanensis*

## Abstract

The complete mitochondrial genome of the *Oreolalax lichuanensis* was determined. It is a circular molecule of 17 702 bp in size and consists of 13 protein-coding genes, 23 tRNA genes, two rRNA genes and a control region (D-loop). The base composition on light strand was 28.0% A, 32.2% T, 24.9% C and 14.9% G. Compared with most other vertebrates, this mitogenome appear a tandem duplication of *tRNA*-*Met* gene. This study will facilitate the further research of the population genetics of this species and systematic study of the genus *Oreolalax*.

*Oreolalax lichuanensis* (Amphibia, Anura, Megophryidae) is known from southwestern China and it generally occurs in the forest land and at mid-altitudes about 1800 m (Fei et al. 2010). We determined the complete mitogenome of this species. The sample of *O. lichuanensis* was obtained from Hubei Province of China. The GenBank accession number of this mitogenome is KU096847.

The complete mitogenome of *O. lichuanensis* is 17 *Oreolalax lichuanensis* 702 bp in length and contains 13 protein-coding genes (PCGs), two ribosomal RNA (rRNA) genes, 23 transfer RNA (tRNA) genes and a control region (D-loop). The base composition of the complete mitochondrial genome was 28.0% A, 32.2% T, 24.9% C and 14.9% G. The A + T base composition (60.2%) was higher than G + C (39.8%), similar to other anurans (Irisarri et al. 2010; Chen et al. 2011; Shi et al. 2012). Except for *ND6* gene and eight tRNA genes (*tRNA-Gln*, *Ala*, *Asn*, *Cys*, *Tyr*, *Ser* (*UCN*), *Glu* and *Pro*) encoded on the L-strand, all other genes were encoded on the H-strand. The *12S* rRNA (933 bp) and *16S* rRNA (1601 bp) genes, locating between *tRNA-Phe* and *tRNA-Leu* genes, were separated by *tRNA-Val* gene. Three types of initiation codons were used for the 13 PCGs, three genes (*COI*, *ND3* and *ND5*) starting with GTG, one starting with CCT (*ND6*), and the rest starting with ATG. Four PCGs (*ATP6*, *COIII*, *ND4* and *Cytb*) ended with an incomplete stop codon T which may be completed by post-transcriptional polyadenylation with poly A tail. *ND5* was the longest gene (1818 bp) and *ATP8* gene (168 bp) was the shortest in 13 PCGs. The 23 tRNA genes with the size ranging from 63 bp to 75 bp were interspersed along the whole genome, and most of the tRNAs formed a colver-leaf structure except the second *tRNA-Ser*, which lost the T ψ C arm. The putative origin of L-strand replication (*O_L_*), with a length of 29 bp between the *tRNA-Asn* and *tRNA-Cys* genes, can fold into a stem loop of secondary structure, similar to other vertebrates (Chen et al. 2011). The D-loop region, which was thought to include the signals for the regulation of mtDNA replication and transcription (Wolstenholme 1992), located between *tRNA-Trp* and *tRNA-Phe*, was 1701 bp in length. This region is heavily biased to A + T nucleotides (63.5%) and we found some repeated sequences in this region. For the whole mitogenome, there were eight regions of gene overlap (ranging from 1 to 10 bp), and eleven intergenic spacer regions (ranging from 1 to 290 bp).

Based on the reported mitogenomes of seven species of Mesobatrachia, four complete mitogenome and three partial mitogenome were used to construct phylogenetic trees. *Pelobates cultripes* was selected as the outgroup. Maximum-likelihood and Bayesian inference methods yielded the same tree topologies. The phylogram acquired from ML methods are shown in Figure 1. The article will provide fundamental data for further investigating the phylogenetic study of the species.

**Figure 1.**
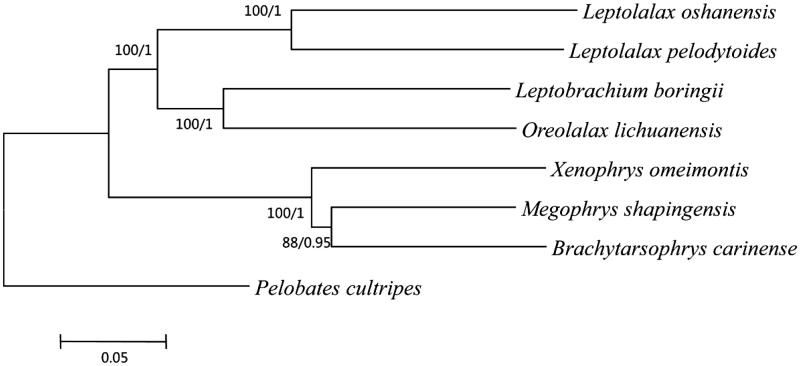
The phylogenetic tree inferred by Phyml 3.0 (Guindon et al. 2010) and MrBayes v3.2.1 (Ronquist et al. 2012). *Leptolalax oshanensis* (KC460337), *Leptolalax pelodytoides* (JX564874), *Leptobrachium boringii* (KJ630505), *Xenophrys omeimontis* (KP728257), *Megophrys shapingensis* (JX458090), *Brachytarsophrys carinense* (JX564854), *Pelobates cultripes* (NC_008144) and *Oreolalax lichuanensis* (KU096847).
